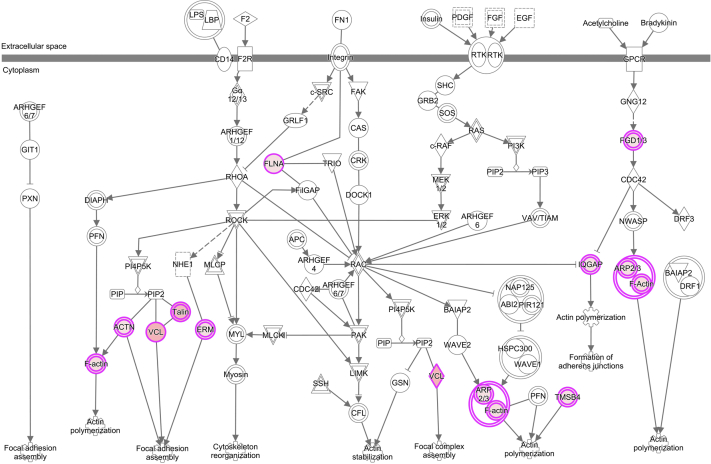# Global Phosphoproteomics Unveils Kinase-Regulated Networks in Systemic Lupus Erythematosus

**DOI:** 10.1016/j.mcpro.2022.100434

**Published:** 2022-10-27

**Authors:** Shuhui Meng, Teng Li, Tingting Wang, Dandan Li, Jieping Chen, Heng Li, Wanxia Cai, Zhipeng Zeng, Dongzhou Liu, Donge Tang, Xiaoping Hong, Yong Dai

**Affiliations:** 1 Clinical Medical Research Center, Guangdong Provincial Engineering Research Center of Autoimmune Disease Precision Medicine, Shenzhen Engineering Research Center of Autoimmune Disease, The Second Clinical Medical College of Jinan University, The First Affiliated Hospital of Southern University of Science and Technology, Shenzhen People’s Hospital, Shenzhen, Guangdong, P. R. China; 2 Department of Rheumatology and Immunology, The Second Clinical Medical College of Jinan University, The First Affiliated Hospital of Southern University of Science and Technology, Shenzhen People’s Hospital, Shenzhen, Guangdong, P. R. China

**Keywords:** phosphoproteomics, SLE remission stage, SLE active stage, kinases, parallel reaction monitoring, CD226, CD226 antigen, GO, Gene Ontology, GSK3B, glycogen synthase kinase-3 beta, HC, healthy control, IKKB, inhibiting kappa B kinase beta, IMAC, immobilized metal-affinity chromatography, IPA, Ingenuity Pathway Analysis, KEGG, Kyoto Encyclopedia of Genes and Genomes, LCP1, plastin-2, LFQ, label-free quantification, MAP3K, mitogen-activated protein kinase kinase kinase, MAP2K, mitogen-activated protein kinase kinase, PBMC, peripheral blood mononuclear cell, PRM, parallel reaction monitoring, RA, rheumatoid arthritis, SLE, systemic lupus erythematosus, SLEDAI, SLE disease activity index, TLN1, talin-1, VCL, vinculin, TGFB1I1, transforming growth factor beta-1-induced transcript 1

## Abstract

Systemic lupus erythematosus (SLE) is an autoimmune disorder characterized by immune complex deposition in multiple organs. Despite the severe symptoms caused by it, the underlying mechanisms of SLE, especially phosphorylation-dependent regulatory networks remain elusive. Herein, by combining high-throughput phosphoproteomics with bioinformatics approaches, we established the global phosphoproteome landscape of the peripheral blood mononuclear cells from a large number of SLE patients, including the remission stage (SLE_S), active stage (SLE_A), rheumatoid arthritis, and healthy controls, and thus a deep mechanistic insight into SLE signaling mechanism was yielded. Phosphorylation upregulation was preferentially in patients with SLE (SLE_S and SLE_A) compared with healthy controls and rheumatoid arthritis populations, resulting in an atypical enrichment in cell adhesion and migration signatures. Several specifically upregulated phosphosites were identified, and the leukocyte transendothelial migration pathway was enriched in the SLE_A group by expression pattern clustering analysis. Phosphosites identified by 4D-label-free quantification unveiled key kinases and kinase-regulated networks in SLE, then further validated by parallel reaction monitoring. Some of these validated phosphosites including vinculin S275, vinculin S579 and transforming growth factor beta-1-induced transcript 1 S68, primarily were phosphorylation of Actin Cytoskeleton -related proteins. Some predicted kinases including MAP3K7, TBK1, IKKβ, and GSK3β, were validated by Western blot using kinases phosphorylation sites-specific antibodies. Taken together, the study has yielded fundamental insights into the phosphosites, kinases, and kinase-regulated networks in SLE. The map of the global phosphoproteomics enables further understanding of this disease and will provide great help for seeking more potential therapeutic targets for SLE.

## Highlights

• Global phosphoproteome landscape were analyzed in PBMCs of SLE patients.
• The phosphoproteomics analysis unveils kinase-regulated networks in SLE.
• Several upregulated phosphosites were validated by PRM in SLE.
• Several core predicted kinases were validated by Western blot in SLE.

## Graphical Abstract

**Figure:**
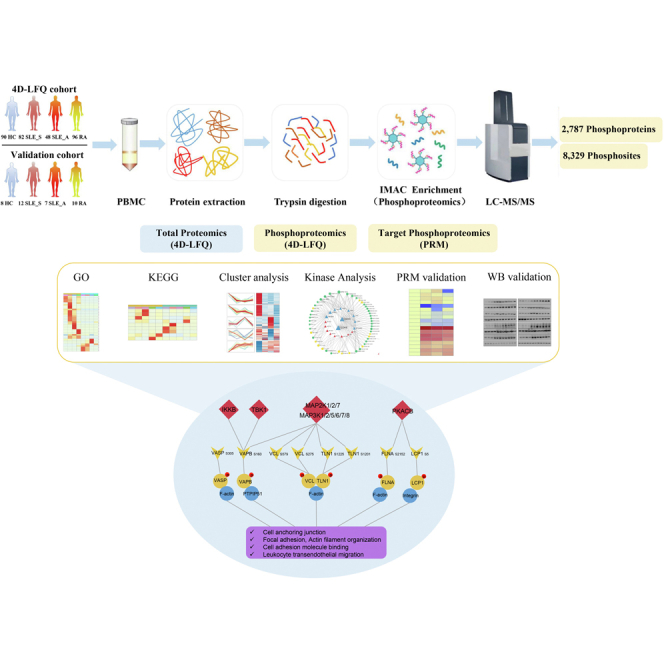

## In Brief

We established the global phosphoproteome landscape of peripheral blood mononuclear cells from healthy controls, systemic lupus erythematosus (SLE) patients (SLE_S, SLE_A), and rheumatoid arthritis by combining high-throughput phosphoproteomics with bioinformatics approaches. We validated some of the key phosphosites and kinases and then built kinase-regulated networks for SLE. The map of the global phosphoproteomic enables further understanding of the pathogenesis of this disease and will provide strategies for seeking potential therapeutic targets for SLE.

Systemic lupus erythematosus (SLE) is a complex systemic autoimmune disease characterized by uncontrolled autoantibody production, immune complex deposition and subsequent organ damage, which could gradually cause severe multisystemic symptoms including lupus nephritis (1). Recent development in high-throughput omics approaches, such as methylomics, proteomics, and single-cell RNA sequencing, has facilitated the pinpointing for key molecular in SLE pathogenesis as well as potential therapeutic targets. However, the underlying molecular mechanism of SLE remains poorly understood, which badly hinders the improvement of SLE treatments (2, 3, 4, 5, 6). Thus, there is an urgent need of revealing the aberrant molecular events in SLE and gaining further insights into its pathogenesis.

Among all the molecular events in cells, protein phosphorylation is one of the most ubiquitous regulatory events participating in signal transduction and is almost involved with every cellular process (7, 8). Dysregulated phosphorylation-mediated cell signaling is present in numerous diseases, including SLE (9). STAT1 serine-727 phosphorylation has been found to play a critical role in B cells *via* promoting autoimmune Ab-forming cell and germinal center responses, thus sequentially stimulating autoantibody production in SLE (10), while the use of kinase inhibitors, such as Bruton tyrosine kinase inhibitors and p38α MAPK kinase inhibitors, showed efficacy in SLE treatment (11, 12). A recent study also suggested that blocking mechanistic target of rapamycin can extend life expectancy by attenuating or preventing SLE (13). Therefore, exploring the global phosphorylation in cells can provide novel and valuable information for identifying reliable diagnostic and therapeutic targets of SLE.

Phosphoproteomics is a novel approach that has exhibited certain advantages through the ability to detect tens of thousands of phosphorylation events with extensive throughput (7). In our study, we present a global phosphoproteomic analysis of peripheral blood mononuclear cells (PBMCs) in patients with SLE_S, SLE_A, rheumatoid arthritis (RA), and healthy controls (HCs) by LC-MS/MS analysis and phosphosite mapping and predict upstream kinases to identify distinct phosphorylation profiles that may result in divergent clinical behaviors, as well as to build kinase-regulated networks. We revealed kinases, phosphosites, and signaling pathways in SLE_S and SLE_A patients that differ from those in HC and RA patients and demonstrated the difference between SLE_S and SLE_A. By presenting abundant information resource to explore phosphorylation events in SLE, our study could provide new insights to elucidating the underlying molecular mechanism of SLE pathogenesis and developing better treatment.

## Experimental Procedures

### Experimental Design and Statistical Rationale

The first cohort consisting of 130 patients with SLE (SLE_S = 82, SLE_A = 48), 96 patients with RA, and 90 HC was used for phosphoproteomics analysis, and the PBMC protein was mixed into 52 pools (14 SLE_S, 7 SLE_A, 16 RA, and 15 HC), and each pool had 1 mg protein that contains approximately six independent samples. The second independent cohort consisting of 19 patients with SLE (SLE_S = 12, SLE_A = 7), 10 patients with RA, and 8 HCs were used for parallel reaction monitoring (PRM) validation, and each sample had 1 mg protein. The third independent cohort consisting of 15 patients with SLE and seven HC was used for Western blot validation. We used RA and HC as the control groups. All three experiments (phosphoproteomics, PRM, and Western blot) were performed with biological replicates. For comparisons between two groups, two-sample two-tailed Student’s *t* tests were performed for phosphoproteomics, a single-tailed, two-sample *t* test for PRM validation, and *p*-value < 0.05 were considered to be significant.

All peripheral blood samples from patients with SLE (SLE_S, SLE_A) and RA were obtained from the Department of rheumatology and immunology, Shenzhen People’s hospital, China. The HCs were recruited from Department of Physical Examination, Shenzhen People’s hospital, China. The present study conformed to the principles of the Declaration of Helsinki. This study was approved by Ethics Committee of the Shenzhen People’s Hospital, China (LL-KY 2019514). And all donors signed a written informed consent to participate in the study. All SLE patients fulfilled The Systemic Lupus International Collaborating Clinics classification criteria (14). The SLE disease activity index (SLEDAI) 2000 is widely used to measure SLE disease activity, and the disease activity score is calculated using the SLEDAI-2K score calculator (https://qxmd.com/calculate/calculator_335/sledai-2k) according to both clinical and laboratory parameters (15). When the SLEDAI score was less than or equal to 4, the SLE patients were considered to be in remission stage SLE_S), while the ones with the SLEDAI score >4 were considered to be in active stage (SLE_A), and the disease activity was assessed using the SLEDAI (16). All RA patients meet the ACR/European League Against Rheumatism 2010 RA classification criteria (17), and the clinical activity of the RA patients was evaluated according to the DAS28 score (18). The clinical information of samples in this study is presented in supplemental Table SX.

### PBMCs Collection

The peripheral blood was collected in EDTA-treated collection tubes from each individual. PBMCs were isolated using density-gradient centrifugation with Ficoll-Hypaque (Invitrogen) according to the manufacturer’s instructions and placed in a −80 °C freezer before for later use. The PBMCs were used for proteomics, phosphoproteomics, phosphosites validation, and kinases validation.

### PBMCs Lysis

Taken out from freezer, the PBMCs were then placed on ice and sonicated using a high intensity ultrasonic processor (Scientz) in lysis buffer (8 M urea, 1% Protease Inhibitor Cocktail). The remaining debris was removed by centrifugation at 12,000*g* at 4 °C for 10 min. At last, the supernatant was transferred to a new tube, and the protein concentration was measured using the BCA kit (Thermo Scientific).

### Trypsin Digestion

The equal amount of protein was taken from each sample. Dithiothreitol was added to the protein solution to a final concentration of 5 mM, and reduction reaction occurred at 56 °C and lasted for 30 min. Then iodine acetamide was added to protein solution to a final concentration of 11 mM, followed by a 15 min-incubation at room temperature in the dark. Then, the protein solution was diluted by adding 100 mM TEAB until urea concentration was less than 2 M. Lastly, trypsin was added at a 1:50 trypsin/protein ratio for the first round of overnight digestion and 1:100 trypsin/protein ratio for the second round of 4 h-digestion.

### Phosphopeptide Enrichment Using Immobilized Metal-Affinity Chromatography

The peptide mixtures were first incubated with immobilized metal-affinity chromatography (IMAC, ThermoFisher Scientific-A32992) microspheres suspension with vibration in loading buffer (50% acetonitrile/6% trifluoroacetic acid). IMAC microspheres with enriched phosphopeptides were then precipitated by centrifugation, and the supernatant was removed. Next, the IMAC microspheres were sequentially washed with 50% acetonitrile/6% trifluoroacetic acid and 30% acetonitrile/0.1% trifluoroacetic acid to remove nonspecifically adsorbed peptides. Then, the enriched phosphopeptides were eluted with elution buffer containing 10% NH4OH on a rotary shaker, and the eluate was collected and dried under vacuum. Finally, enriched phosphopeptides were desalted using C18 ZipTips (Millipore) and freeze-dried under vacuum for LC-MS/MS analysis.

### LC-MS/MS Analysis for Phosphoproteomics

The peptides were dissolved in solvent A (0.1% formic acid, 2% acetonitrile in water) of liquid chromatography mobile phase, directly loaded onto a home-made reversed-phase analytical column (25-cm length, 100 μm i.d.) packed with 1.9 μm/120 Å ReproSilPurC18 resins (Dr Maisch GmbH). Solvent B was 0.1% formic acid in acetonitrile. Liquid phase gradient was set as follows:0 to 50 min, 2% ∼ 22%B; 50 to 52 min, 22% ∼ 35%B; 52 to 55 min, 35% ∼ 90%B; and 55 to 60 min, 90%B; All peptides were separated at a constant flow rate of 450 nl/min by online nanoElute UHPLC system (Bruker Daltonics).

The peptides were subjected to capillary source followed by the timsTOF Pro (Bruker Daltonics) mass spectrometry. The electrospray voltage was set to 1.6 kV. Precursors and fragments of peptide were detected and analyzed by TOF. The MS/MS scan range was from 100 to 1700 m/z. Parallel accumulation serial fragmentation mode was used for data acquisition. Precursors with charge states of 0 to five were selected for fragmentation, and 10 parallel accumulation serial fragmentation-MS/MS scans were acquired per cycle. The dynamic exclusion time of tandem mass spectrometry was set to 30 s to avoid repeated scanning of precursors.

### Database Search

The LC-MS/MS data were analyzed by the MaxQuant search engine (v.1.6.6.0). The retrieval parameters were set as follows: tandem mass spectra were searched against the human SwissProt database (Homo_sapiens_9606_SP_20191115, 20,380 entries) concatenated with reverse decoy database; the enzyme specificity was trypsin/P, and two missing cleavages were permitted; the minimum length of peptide was set to seven amino acid residues, and the maximum modification number was set to five; the mass tolerance for precursor ions was set as 20 ppm in both first search and main search, and the mass tolerance for fragment ions was set to 0.02 Da; Carbamidomethyl on Cys was set to fixed modification, and acetylation on protein N-terminal, oxidation on Met, and phosphorylation on Ser, Thr, and Tyr were set as variable modifications. FDR was adjusted to <1%.

### Phosphoproteomics Data Processing

The signal intensity of each peptide in different samples was calculated as follow: The relative quantitative value © of the modified peptide in different samples was obtained after the signal intensity value (I) was transformed by the center. The formula for calculation was as follows: Rij = Iij/Mean(Ij)Rij = Iij/Mean(Ij), I represented the samples, and J represented the peptides. The relative quantitative value of the modified site was divided by the relative quantitative value of the protein corresponding to the modified site to eliminate the influence of protein expression on the modified expression. The quantitative value was log2 transformed to ensure that they were normally distributed, then the significance of *p*-value is calculated using a two-sample, two-tail Student’s *t* test in two groups. Phosphosites were considered significantly differentially expressed if the *p*-value was <0.05 and absolute fold change was ≥1.5

### Pathway Analysis

#### Enrichment of Gene Ontology Analysis

Phosphoproteins were classified by Gene Ontology (GO) annotation (http://www.ebi.ac.uk/GOA/) into three categories: biological process, cellular compartment, and molecular function. The Kyoto Encyclopedia of Genes and Genomes (KEGG) database was used to annotate protein pathway, KEGG online service tool KAAS (http://www.genome.jp/kaas-bin/kaas_main) was used to annotated protein’s KEGG database description, and then the annotation result was mapped on the KEGG pathway database using KEGG online service tools KEGG mapper (http://www.kegg.jp/kegg/mapper.html). The functional enrichment of GO and KEGG was analyzed by a two-tailed Fisher’s exact test to test the enrichment of the differentially modified protein against all identified proteins. When the *p*-value was less than 0.05, the pathway was considered significant. Additionally, we submitted differentially expressed phosphoproteins to Ingenuity Pathway Analysis (IPA) software (Qiagen) for core pathways analysis. The Z scores were calculated based on Ingenuity Knowledge Base and predicted the activation status of each pathway. The higher the Z score is, the more activated this pathway is, and the lower the Z value is, the more inhibited this pathway is. Fisher’s exact test was utilized to calculate *p*-values with IPA, with *p*-value < 0.05 considered as significant.

#### Expression Pattern Clustering Analysis

The Mfuzz method was used to perform cluster analysis for modification site abundance shift under different continuous samples. A new clustering algorithm, the fuzzy c-means algorithm, was used. We first transformed the relative expression level of the modified sites by Log2, then screened out the modified sites with SD > 0.5. After screening, the remaining modification sites were used for expression pattern clustering analysis by the Mfuzz method. These modified sites were then divided into five clusters, and the functional enrichment of GO, KEGG, and domains were performed for each cluster. The analysis method was Fisher’s exact test. It was considered significant when the *p*-value was less than 0.05.

#### Motif Analysis

The software MOMO (motif-x algorithm) was used to analyze the motif characteristics of phosphosites. When the number of phosphosites in a characteristic sequence form was more than 20, and the *p*-value of statistical test was less than 0.000001, the characteristic sequence form can be considered as a motif for phosphosites.

#### Kinase Prediction and Kinase-Substrate Regulatory Network Analysis

The iGPS1.0 software (http://igps.biocuckoo.org/) was used to predict upstream phosphokinase, which was based on the theory of short linear motifs around phosphosite (p-sites) to provide the primary specificity. The kinase activity was predicted by the GSEA method. The two types of GSEA input data were the corresponding phosphosites of each kinase, as.gmt file, and the ratio value of the kinase in each comparison group as the expression profile.gct file. The bar graphs were analyzed by ggplot2 R package. The predicted kinases with positive or negative activity and significantly differential expressed phosphosites were used to construct a kinase-substrate regulatory network by Cytoscape.

#### Kinase-Pathway Network Analysis

The kinase-pathway network analysis was conducted based on the predicted results of kinase activity in the three comparison groups (SLE_S *versus* HC, SLE_A *versus* HC, and SLE_A *versus* SLE_S), and the upregulated and downregulated kinases (top10) were selected for the relationship network analysis. Next, when choosing kinases, consider whether they catalyze differentially expressed phosphosites (Fold change >1.5, *p*-value <0.05), if not, skip the kinase. Then, based on the proteins of the phosphorylation sites catalyzed by these kinases, the KEGG pathway and GO functional annotation results were selected (*p*-value <0.05). Finally, a “differential kinases-phosphosites-phosphoproteins-pathways” quadripartite network was constructed that also called kinase-pathway network. Kinase-pathway network was visualized by Cytoscape software.

#### Validation Studies Using PRM

In this study, we applied tier 3 PRM analyses. As tier 3 measurements were usually considered as powerful methods especially in early-stage biological studies, which enable repeatable measurement of the same sets of analytes across experiments but do not employ internal standards for either accurate or precise measurement of the levels of each analyte. Tier 3 methods do not constitute assays but instead employ targeted strategies to bias toward detection of a predefined set of analytes.

The processes of protein extraction, trypsin digestion, and phosphopeptide enrichment are consistent with phosphoproteomics. The peptides were dissolved in solvent A (0.1% formic acid, 2% acetonitrile in water), directly loaded onto a home-made reversed-phase analytical column (25-cm length, 100 μm i.d.). Solvent B was 0.1% formic acid in 90% acetonitrile. Liquid phase gradient was set as follows: 0∼40 min，3% to 17%B; 40∼52 min，17% to 28% B; 52∼56 min，28% to 80% B; 56∼60 min，80%. All peptides was separated at a constant flow rate of 500 nl/min on an EASY-nLC 1200 UPLC system (Thermo).

The peptides were subjected to NSI source followed by MS/MS in Q ExactiveTM plus (Thermo) coupled online to the UPLC. The electrospray voltage was set to 2.1 kV. The m/z scan range was 490 to 1300 for full scan, and intact peptides were detected in the Orbitrap at a resolution of 35,000. Peptides were then selected for MS/MS, and the normalized collisional energy was set to 27, and the Orbitrap scan resolution was set to 17,500. The automatic gain control was set at 3E6, and the maximum IT was set at 200 ms for full MS; The automatic gain control was set at 1E5 and the maximum IT was set at 200 ms for MS/MS, and the isolation window for MS/MS was set at 1.6 m/z.

The MS/MS data were processed using Skyline (v.4.1.0.18166). The data were analyzed by the MaxQuant search engine (v.1.5.2.8). Phosphopeptide settings were as follows: Tandem mass spectra were searched against the human SwissProt database (Homo_sapiens_9606_SP_20191115, 20,380 entries) concatenated with reverse decoy database. The enzyme was set as trypsin [KR/P], max missed cleavage was set as 3. The peptide length was set as 7 to 32, Variable modification was set as carbamidomethyl on Cys and oxidation on Met, and max variable modifications were set as 3. Transition settings were set as follows: precursor charges were set as 2, 3, ion charges were set as 1, ion types were set as b, y. The product ions were set as from ion three to last ion. The ion match tolerance was set as 0.02 Da.

#### Western Blot Analysis

For whole cell–protein extraction, the PBMC was pelleted and lysed in RIPA buffer (50 mM Tris-HCl, pH 8.0, 150 mM NaCl, 1% Triton-X100, 1% sodium deoxycholate, 0.1% SDS, and 1 mM EDTA) supplemented with proteinase inhibitors (Sigma) on ice for 15 min. After protein concentration quantification by BCA kit (Sigma), same amount of proteins were calculated and an equal volume of 2× sample buffer (950 μl of Laemmli buffer +50 μl β-mercaptoethanol) was added, and the lysates were then boiled for 10 min at 99 °C. For Western blot analysis, the boiled samples were separated by SDS-PAGE, then transferred to nitrocellulose membranes (Millipore). The membranes were blocked with 3% non-fat milk at room temperature for 1 h and washed with TBST before incubating with specific primary antibodies overnight at 4 °C. Antibodies for IKKβ (CST #8943), p-IKKβ Ser176/180 (CST #2697), TBK1 (CST #3504), p-TBK1 Ser172 (CST #5483), and p-GSK3β Ser9 (CST #5558) were purchased from Cell Signaling Technology, antibody for MAP3K7 (PTM-5764) was purchased from PTM Biolabs, antibodies for p-MAP3K7 Thr187 (28958-1-AP) and β-actin (20536-1-AP) were purchased from Proteintech. The membranes were then washed with TBST at room temperature for 3 times followed by incubating with secondary antibodies at RT for 1 h, and the membranes were washed for 3 times and incubated with ECL (ThermoFisher) to collect the data.

## Results

### Generation of Phosphoproteomic Data Obtained From PBMCs

**Fig. 1:**
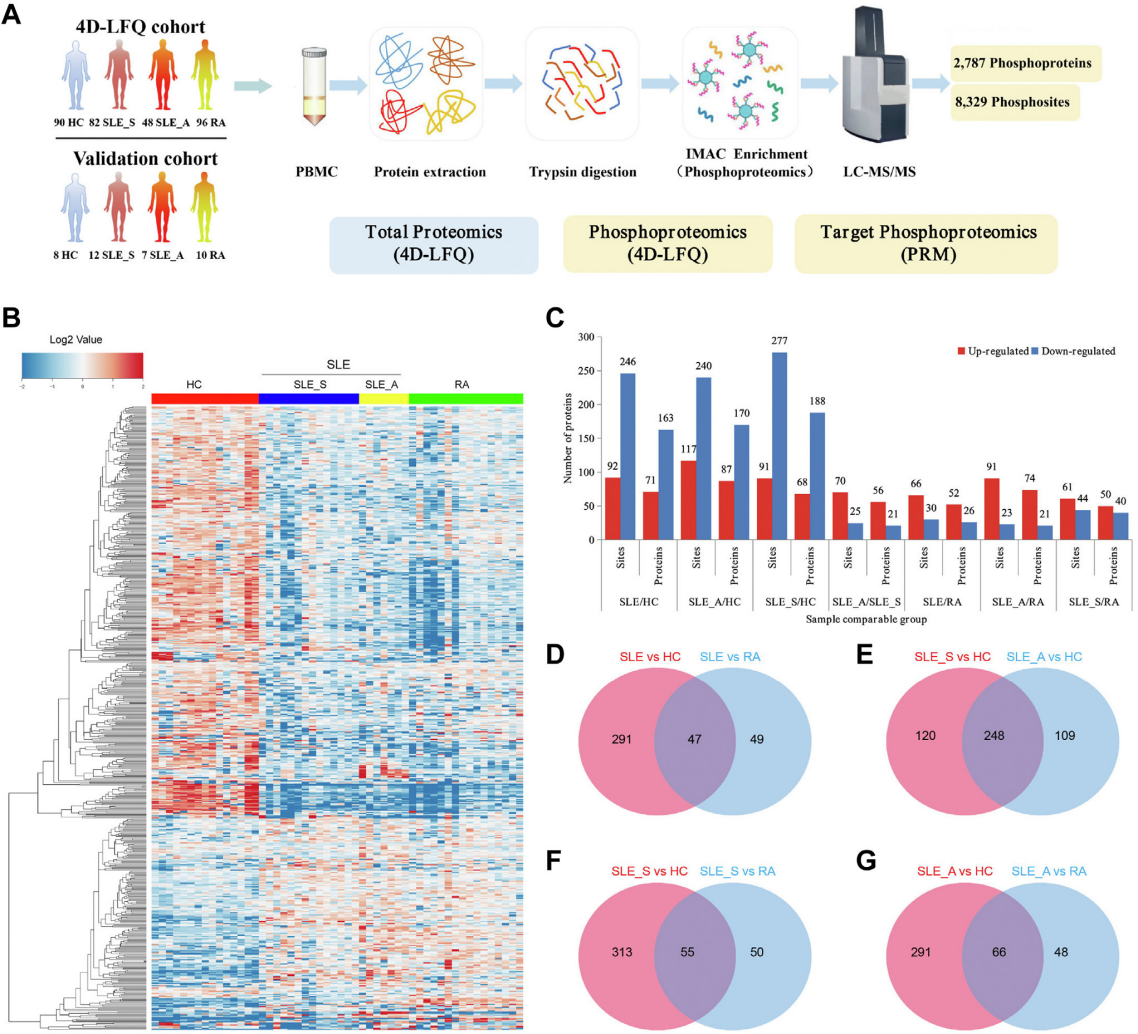
**Overview of the high-throughput phosphoproteomics**. *A*, PBMCs from three experimental groups, including patients with SLE (SLE_S = 82, SLE_A =48) (n = 130), RA (n = 96), and HC (n = 90) were subjected to LC-MS/MS analysis with a 4D-LFQ approach to generate phosphoproteomic datasets and bioinformatics analysis. *B*, hierarchical clustering of the differentially expressed phosphosites in patients with SLE (SLE_S, SLE_A), HC, and RA. *C*, the histogram of significantly regulated phosphosites on phosphorylated proteins in seven comparable groups, including SLE *versus* HC, SLE_ *versus* RA, SLE_S *versus* HC, SLE_A *versus* HC, SLE_A *versus* SLE_S, SLE_S *versus* RA, and SLE_A *versus* RA. Differential phosphosite expression was defined as fold change >1.5, and the *p*-value <0.05. *D–G*, Venn diagram showing unique and overlapping phosphosites in different comparison groups. HC, healthy control; IMAC, immobilized metal-affinity chromatography; PBMC, peripheral blood mononuclear cell; RA, rheumatoid arthritis; SLE, systemic lupus erythematosus; LFQ, label-free quantification.

To characterize the phosphorylation events of patients with SLE (SLE_S (SLEDAI score ≤4) and SLE_A (SLEDAI score >4), we used RA and HC as the control groups in the analysis and performed LC-MS/MS with a 4D-label-free quantification (LFQ) approach to generate phosphoproteomic datasets (Fig. 1*A* and supplemental Table SA). As depicted in Fig. 1*A*, we quantified 8329 phosphosites on 2787 phosphorylated proteins and validated target phosphosites using PRM. Hierarchical clustering analysis showed the differentially expressed phosphosites in patients with SLE, RA, and HC (Fig. 1*B*). We counted differentially expressed phosphosites on proteins in patients with SLE compared with HC and patients with RA (Fig. 1*C* and supplemental Table S1) and found that 291 phosphosites are specifically expressed in SLE when compared with HC, while 49 phosphosites are specifically expressed in SLE when compared with RA. This result suggested more differentially expressed phosphosites are shown in SLE when compared to HC than when compared to RA (Fig. 1*D* and supplemental Table S2). We also observed more significant differences in these differentially expressed phosphosites when SLE_S and SLE_A were compared to HC than in those differentially expressed phosphosites from comparison between SLE_S and SLE_A *versus* RA (Fig. 1, *F* and *G* and supplemental Table S2). When both compared with HC, we found many common differentially expressed phosphosites in SLE_S and SLE_A (Fig. 1*E* and supplemental Table S2).

### High-Resolution Overview of Signaling Pathways Revealed in SLE

**Fig. 2:**
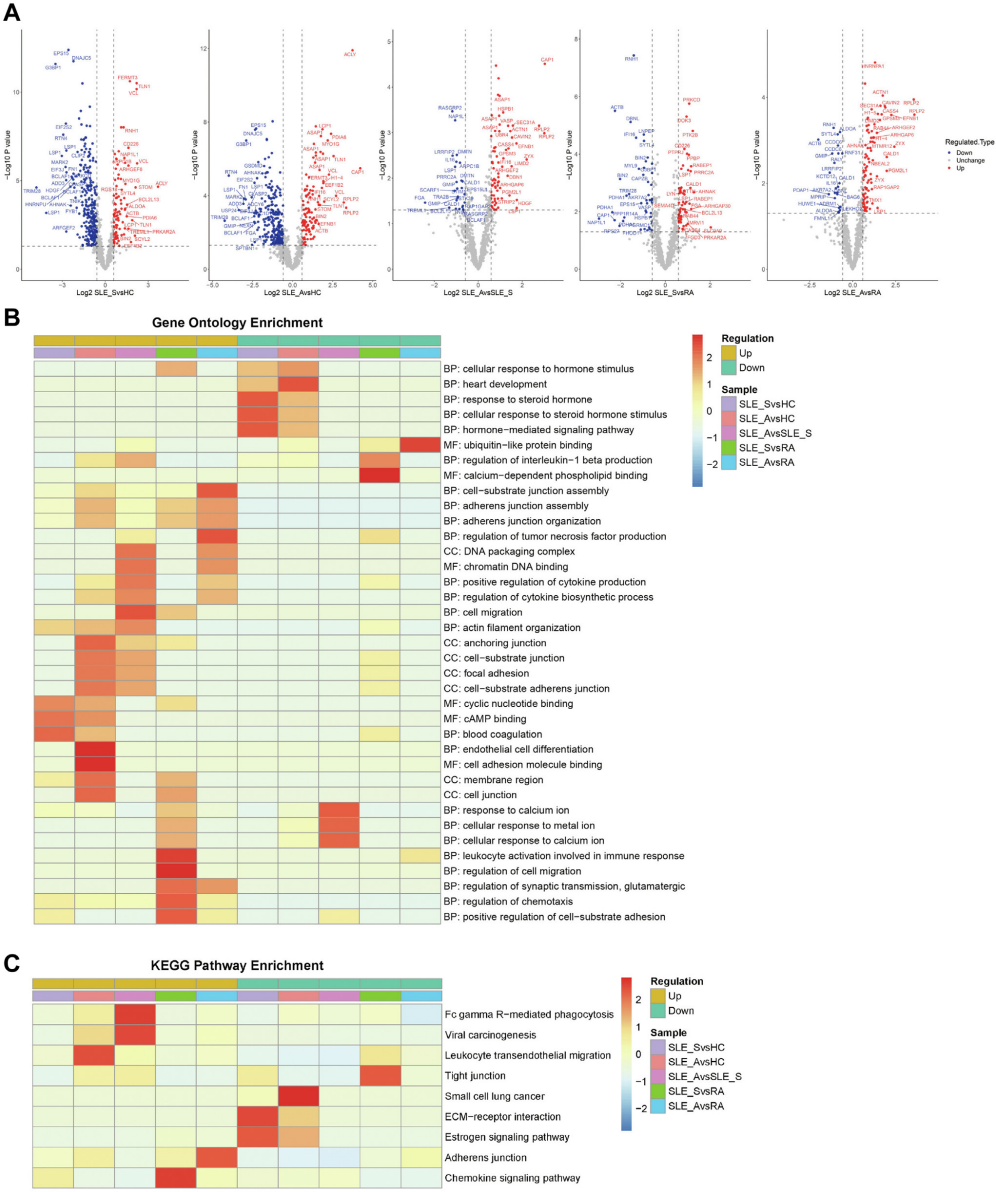
***Top* enriched pathway profiles of GO and KEGG**. *A*, volcano plots of quantitative analysis of phosphosites were shown in five comparable groups (Fold change >1.5, *p*-value <0.05). Significant upregulated sites were indicated in *red* and downregulated ones in *blue*, with *top* 25 sites were highlighted. *B* and *C*, the *top* 30 GO terms and *top* three KEGG pathways for significantly changing phosphosites in comparable groups, which was divided into two sets: upregulated (*left*) and downregulated (*right*). The following criteria was applied for GO terms and KEGG pathways: *p*-value <0.05 was considered as significant. The color represented the degree of enrichment for GO terms and KEGG pathways, *red* represented strong enrichment and *blue* represented weak enrichment. GO, Gene Ontology; HC, healthy control; KEGG, Kyoto Encyclopedia of Genes and Genomes; RA, rheumatoid arthritis; SLE, systemic lupus erythematosus.

Next, we primarily analyzed the subgroups of SLE to explore the differences of functions in patients with SLE_S and SLE_A compared with HC and those with RA. Additionally, we also compared the differences of functions between SLE_A and SLE_S. Firstly, the volcano plot showed top 25 upregulated and downregulated phosphosites in patients with SLE_S and SLE_A compared to HC and those with RA and SLE_A *versus* SLE_S (Fig. 2*A*). Based on these differences, a GO enrichment analysis of phosphoprotein was performed, and the results suggested that the upregulated phosphoproteins in the SLE_S and SLE_A groups when compared with HC and those with RA tend to be involved in executive functions, including actin filament organization, adherens junction organization, and cell−substrate junction assembly, and the SLE_A group showed a higher degree of pathway enrichment than the SLE_S group (Fig. 2*B* and supplemental Table S3). Compared with the SLE_S group, phosphoproteins involved in focal adhesion, cell migration, and positive regulation of cytokine production were enriched in the SLE_A group. These functions suggest that these phosphoproteins may contribute to disease severity in SLE_A (Fig. 2*B* and supplemental Table S3). Chemotaxis in the SLE (SLE_S and SLE_A) group was upregulated when compared with RA group. The most frequently obtained GO terms of enrichment associated with downregulated phosphoproteins were related to heart development in patients with SLE_S and SLE_A (Fig. 2*B* and supplemental Table S3). In addition, we performed KEGG analysis of differentially expressed phosphoproteins. The most significantly upregulated pathway in patients with SLE_A was that of leukocyte transendothelial migration, with increased phosphorylation of vinculin (VCL), VASP, ACTN1, ACTB, F11R, and PTK2B (Fig. 2*C* and supplemental Table S4). Compared with those in the SLE_S group, the phosphoproteins involved in Fc gamma R−mediated phagocytosis were significantly enriched in the SLE_A group, and this function was regulated primarily by the phosphorylation of ASAP1, SYK, and VASP. The most frequently obtained terms associated with enriched upregulated phosphoproteins in the SLE_S group were the chemokine signaling pathway and in the SLE_A group were adherens junctions, compared with the RA group (Fig. 2*C* and supplemental Table S4). Together, these results pinpointed distinct pathways and associated phosphoproteins in patients with SLE_S and SLE_A compared with those in HC and RA patients, which later would be meaningful in further mechanistic exploration.

We identified 45 differentially regulated phosphoproteins based on the results of GO and KEGG pathway analyses in SLE (Fig. 2, *B*, *C* and supplemental Table S8). To further elucidate underlying function of these phosphoproteins, we performed pathway analysis using IPA software. We presented top four activated signaling pathways that was similar to GO and KEGG results, such as actin cytoskeleton signaling, leukocyte extravasation signaling, and integrin signaling, which were crucial process for cell migration (supplemental Fig. S1, *A* and *B*). Our data also showed that the actin cytoskeleton signaling, one of these activated signaling pathways, regulated by talin-1 (TLN1), VCL, FLNA, and so on (supplemental Fig. S1*C*). These results suggest that the identified phosphoproteins facilitate actin polymerization and focal adhesion assembly.

### Expression Pattern Clustering Identified Five Distinct Patterns of Phosphosites Expression

**Fig. 3:**
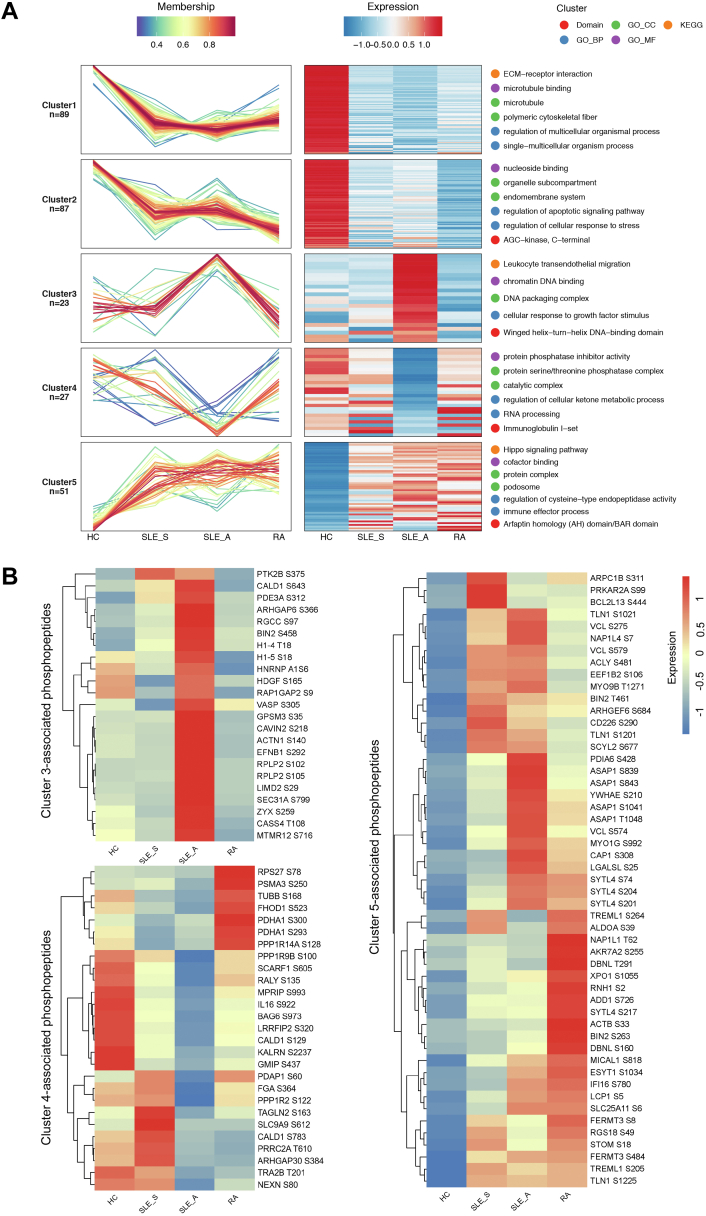
**Expression pattern clustering and functional analysis of phosphosites**. *A*, five phosphoproteomic clusters were identified using the Mfuzz method. Line chart of phosphosite expression level (*left*), the horizontal coordinate represented samples (HC, SLE_S, SLE_A, and RA). The *vertical* coordinate represented the relative expression level of the phosphosites. Each line represented a phosphosite and was color-coded by the cluster membership. Heatmap of expression level (*center*), the horizontal coordinate represented samples (HC, SLE_S, SLE_A and RA), and the vertical coordinate represented different phosphosites, the color of heatmap indicated the relative expression of the phosphosites in the sample. Pathway enrichment analysis (*right*), Gene Ontology (GO), KEGG, and domain. The GO mainly included three aspects: biological process (BP), cellular component (CC), and molecular function (MF), and the *top* two significantly enriched items were presented in each function by different colors, *p*-value <0.05. *B*, the heatmap showed the specific phosphosites in cluster 3, 4, and 5. HC, healthy control; KEGG, Kyoto Encyclopedia of Genes and Genomes; RA, rheumatoid arthritis; SLE, systemic lupus erythematosus.

To select phosphosites with significant changes in abundance, the relative expression of phosphosites was calculated by log2 transformation. The phosphosites that met the SD>0.5 criterion was selected. Finally, 277 phosphosites were grouped into five distinct clusters by the Mfuzz method, and a functional enrichment analysis was performed for each cluster. Among these clusters, clusters 1 and 2 represented phosphosites downregulated in SLE_S, SLE_A, and RA. For the phosphoproteins in cluster 1, the KEGG analysis showed significant downregulation in ECM–receptor interaction, and the GO enrichment analysis showed significant downregulation in microtubule binding, regulation of the multicellular organismal process, and polymeric cytoskeletal fibers. Cluster 2 was enriched in phosphoproteins involved in the regulation of the apoptotic signaling pathway, regulation of the cellular response to stress, nucleoside binding, and organelle subcompartments. Cluster 4 included 27 specifically downregulated phosphosites in the SLE_A group that are associated with protein phosphatase inhibitor activity, catalytic complex, regulation of cellular ketone metabolic process, and RNA processing (Fig. 3*A* and supplemental Table S5).

Cluster 3 included 23 specifically upregulated phosphosites in the SLE_A group, and they were enriched in leukocyte transendothelial migration, which is an important step in driving inflammatory immune responses and is regulated primarily by S305 phosphorylation of VASP, S 375 phosphorylation of PTK2B, and S140 phosphorylation of ACTN1 (Fig. 3, *A* and *B*). Cluster 3 phosphoproteins were also involved in altered DNA function, including the DNA packaging complex, chromatin DNA binding, and winged helix-turn-helix DNA-binding domain, which are regulated by T18 phosphorylation of histone H1.4 and S18 phosphorylation of histone H1.5. Cluster 5 included 57 upregulated phosphosites among the three groups of patients, with SLE_S, SLE_A, and RA, and they were enriched in the Hippo signaling pathway, regulation of cysteine−type endopeptidase activity, and the immune effector process, among other functions and components. Several commonly upregulated phosphosites were found in the SLE_S and SLE_A groups, including phosphorylated CD226 antigen (CD226), VCL, and TLN1. Additionally, cluster 5 included multiple upregulated phosphosites of ASAP1 in the SLE_A group (Fig. 3, *A* and *B*). Taken together, the overall results of the phosphoproteomic analysis indicate that there are multiple signaling events contributing to the regulatory control in SLE progression.

### Phosphoproteomics Identified Key Kinases and Kinase-Regulated Networks in SLE

**Fig. 4:**
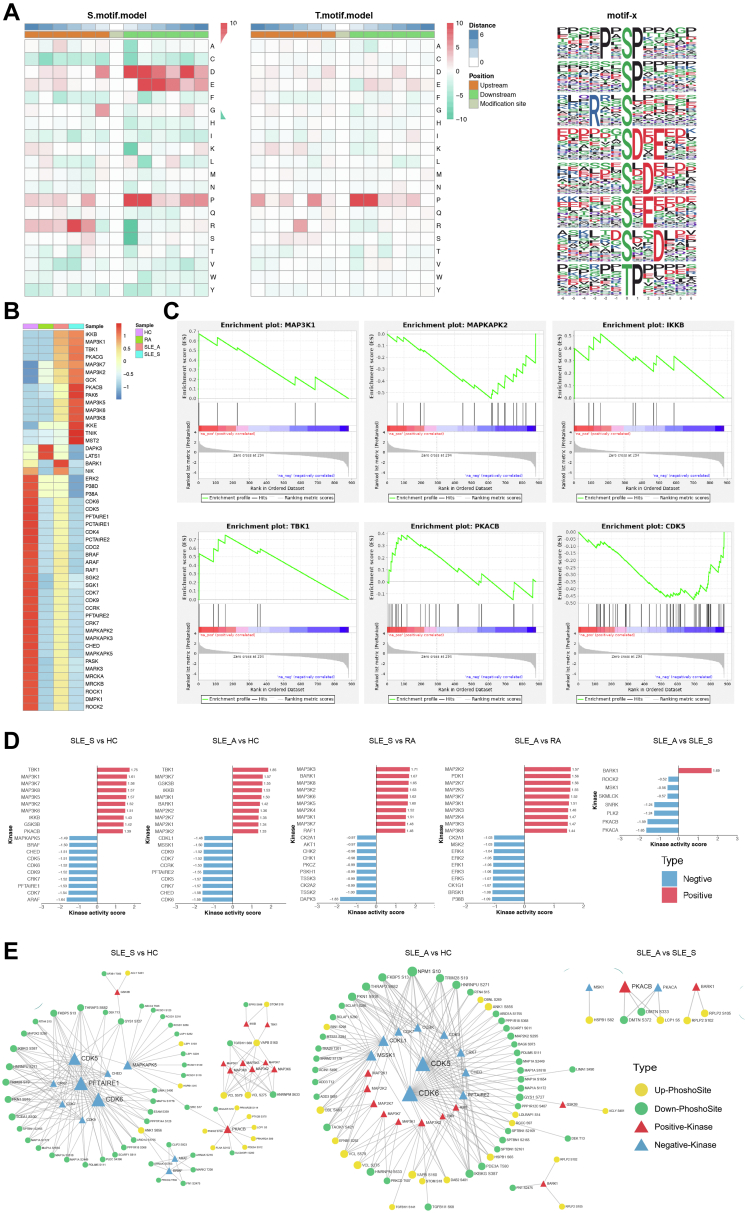
**Kinase predicted and kinase-pathway network analysis**. *A*, significant enriched phosphorylation motifs extracted from the overrepresented phosphopeptide dataset by motif-X. The motifs were from phosphoserine and phosphothreonine. *B*, the *top* 50 kinases were listed for four groups (HC, SLE_S, SLE_A, and RA) according to the absolute value of normalized enrichment score (NES). *C*, NESs of several kinases for SLE_S *versus* HC were shown, each substrate (phosphopeptide) was represented as a *vertical black line*. *D*, the *top* 10 kinase activity scores were displayed for five comparable groups, including SLE_S *versus* HC, SLE_A *versus* HC, SLE_A *versus* SLE_S, SLE_S *versus* RA, and SLE_A *versus* RA. *E*, integrative networks of the kinase-phosphosite interaction were analyzed for three comparable groups, including SLE_S *versus* HC, SLE_A *versus* HC, and SLE_A *versus* SLE_S. The relationship between differentially modified protein s and kinases significantly enriched by GSEA was screened, and Cytoscape software was used for drawing. HC, healthy control; RA, rheumatoid arthritis; SLE, systemic lupus erythematosus.

Through a motif analysis, we found that the amino acids aspartate (D), glutamate (E)©, and proline (P) were preferentially located at upstream of serine phosphosites, and proline (P) was preferentially present at upstream of threonine phosphosites (Fig. 4*A*). We further used iGPS1.0 software to predict the upstream phosphokinases based on the phosphosites, and each of these kinases contained at least one regulated phosphosite. Then, we used the GSEA method to predict kinase activity, and the normalized enrichment scores of the enrichment results were regarded as kinase activity scores. There were apparent differences in patients with SLE_S and SLE_A compared with HC and patients with RA, and there were high similarities between SLE_S and SLE_A patients (Fig. 4*B* and supplemental Table S6). Most of the kinases predicted to be strongly activated included several members of the mitogen-activated protein kinase kinase kinase (MAP3K) family, including MAP3K1, MAP3K2, and MAP3K7, in patients with SLE_S and SLE_A compared with HC and those with RA. Several members of the mitogen-activated protein kinase kinase (MAP2K) family were also activated, including MAP2K1, MAP2K2, and MAP2K7, in patients with SLE_A compared with HC and those with RA. Besides, TBK1, inhibiting kappa B kinase beta (IKKB) and glycogen synthase kinase-3 beta (GSK3B) are activated in SLE_S and SLE_A patients when compared with HC. The activity showed an increase for Beta-adrenergic receptor kinase 1 in SLE_A compared to SLE_S patients. The kinases predicted to be commonly downregulated in patients with SLE_S and SLE_A included several cell cycle kinases (CDK5, CDK6, CDK7, CDK9 CRK7, and CHED), which are activated by binding to a cyclin and mediate progression through the cell cycle (Fig. 4, *C* and *D*).

Next, we constructed kinase–substrate interaction networks and retrieved the kinase–substrate relationship from iGPS for the SLE groups. The interaction networks revealed that the upregulated kinase activity of MAP3Ks, MAP2Ks, TBK1, and IKKB was directly linked to increased phosphorylation substrates such as VCL S275, VCL S579, VAPB S160, and decreased transforming growth factor beta-1-induced transcript 1 (TGFB1I1) S68 in SLE_S and SLE_A groups. The increased phosphosite of ACLY S481 was associated with the upregulated kinase activity of GSK3B in the SLE_S and SLE_A groups (Fig. 4*E*). The relationship of these kinases and phosphorylation substrates provided clues for the regulation mechanism of SLE.

**Fig. 5:**
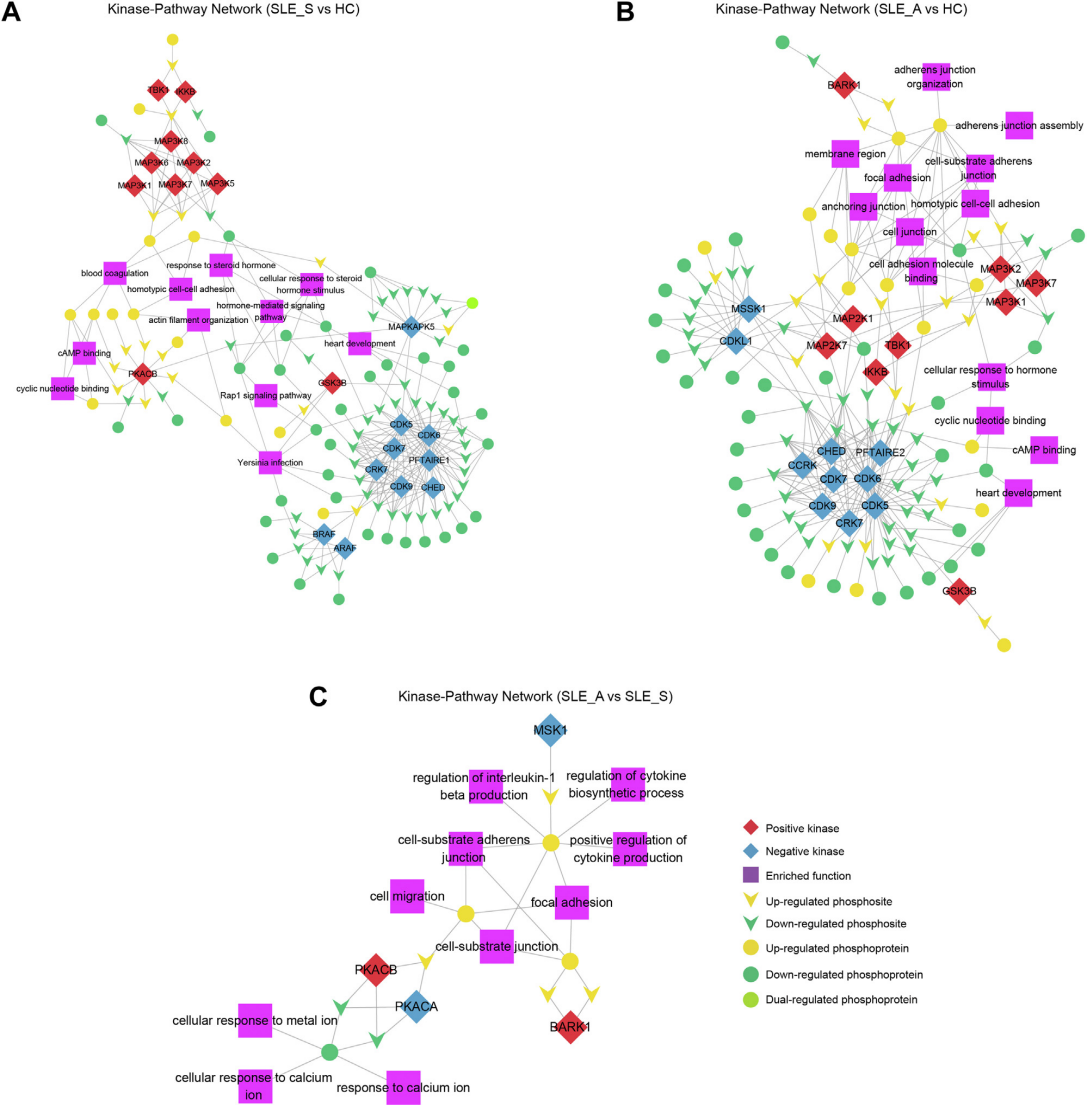
**Integrative networks of the kinase-pathway interaction were analyzed for three comparable groups**. *A*, SLE_S *versus* HC; *B*, SLE_A *versus* HC; *C*, SLE_A *versus* SLE_S. Kinase-function network was visualized by Cytoscape software, positive kinases represented by *red* and negative kinases by *blue*. *Yellow* represented upregulated phosphosites/proteins, and *green* represented downregulated phosphosites/proteins. In addition, *yellow-green* meant there were both upregulated and downregulated phosphosites in a protein, and *purple squares* represented enriched function (KEGG and GO). HC, healthy control; SLE, systemic lupus erythematosus.

Based on the kinase–phosphosite correlation suggested by our data, next, we wanted to understand how the phosphoproteomic signaling pathways interact with kinases, and therefore, we performed a kinase-pathway network analysis. We found that upregulated pathways (homotypic cell–cell adhesion, actin filament organization, and blood coagulation) were correlated with upregulated kinases (MAP3Ks, TBK1, IKKB, and PKACB) in the SLE_S group. In SLE_A group, several upregulated pathways, including those involved with focal adhesion, anchoring junctions, and cell–substrate adherens junctions were also correlated with upregulated kinases, such as MAP3Ks, MAP2Ks, TBK1, and IKKB. This analysis suggests that phosphoproteomic signaling pathways are linked with and regulated by the predicted kinases (Fig. 5).

### Validation of Phosphosites and Phosphokinases Identified by MS Using PRM and Western Blot in PBMC

**Fig. 6:**
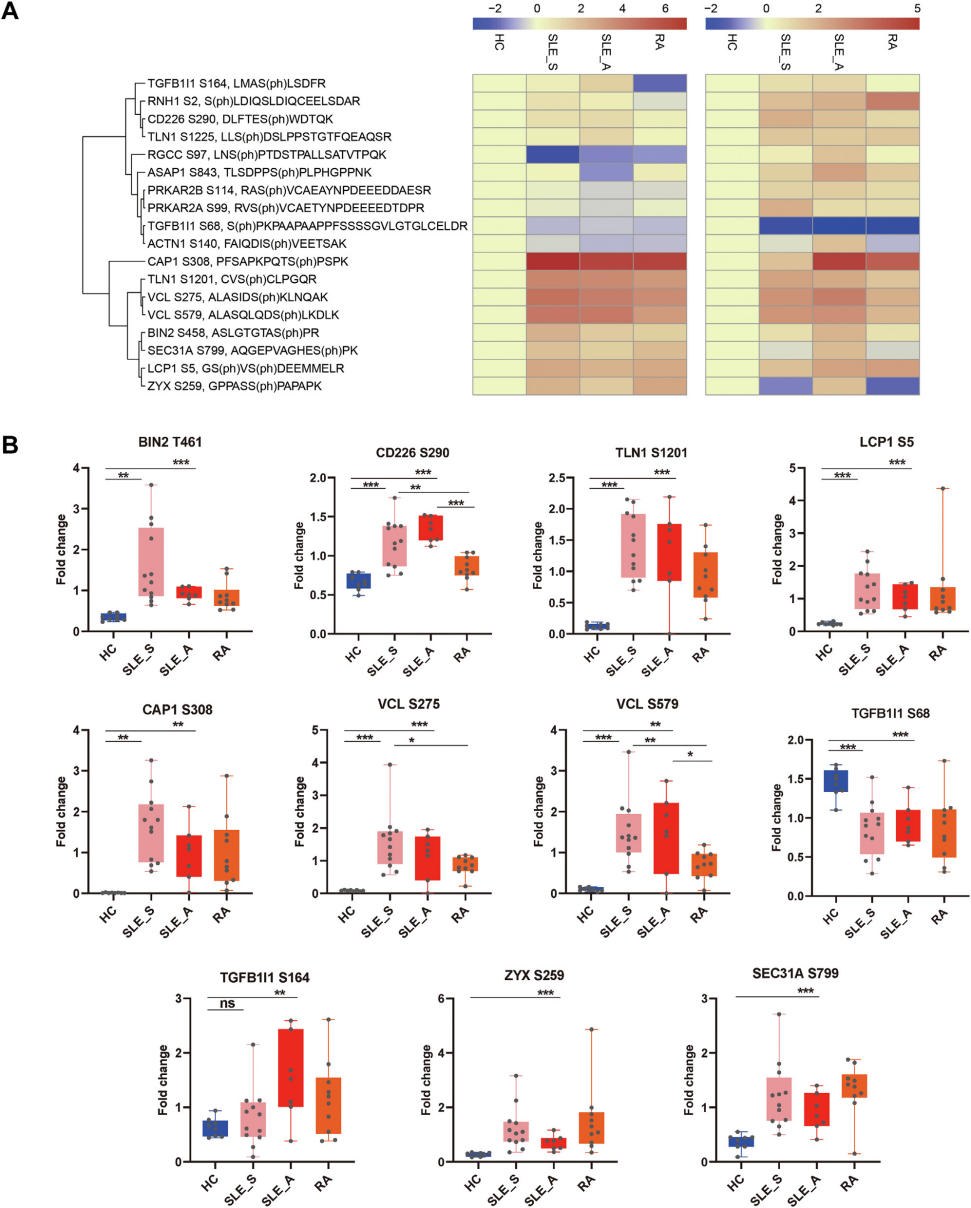
**Parallel reaction monitoring (PRM) validation**. *A*, heatmap showed 18 quantified phosphosites. The PRM (*left*) and 4D-LFQ data (*right*) were depicted, the phosphosites were clustered according to the PRM profiles, the PRM data were log2-transformed, and the color code represented log2-fold changes in SLE_S, SLE_A, and RA compared with healthy controls (log2-ratios were ranged from −2 to 6). *B*, validated phosphosites were shown that has the same trend with 4D-LFQ. Note: ∗,∗∗,∗∗∗ represent *p*-value <0.05, <0.01, <0.001, respectively. ns, nonsignificant; LFQ, label-free quantification; RA, rheumatoid arthritis; SLE, systemic lupus erythematosus.

To validate the 4D-LFQ data, we quantified 18 phosphosites in the four different groups by PRM (supplemental Table SB). We compared PRM data with those from our 4D-LFQ analysis and found that for most phosphosites, the data showed good correlation (Fig. 6*A* and supplemental Table S7). The phosphosites showed the same trend, although the fold change varied notably between the results generated through two techniques. Seven phosphosites (BIN2 S458, CD226 S290, TLN1 S1201, plastin-2 [LCP1] S5, adenylyl cyclase-associated protein 1 [CAP1] S308, VCL S275 and VCL S579) were upregulated, and one phosphosite was downregulated (TGFB1I1 S68) in the SLE_S and SLE_A groups compared with the HC group, and among these phosphosites, CD226 S290, VCL S275, and VCL S579 showed higher expression in the SLE_S and SLE_A groups than in the RA group (Fig. 6*B*). Three additional phosphosites (TGFB1I1 S164, ZYX S259 and SEC31A S799) were confirmed to be upregulated in SLE_A group compared with the HC group (Fig. 6*B*). Nevertheless, using PRM, we failed to validate the high expression of ASAP1 S843, SEC31A S799, ACTN1 S140, or RGCC S97 in the SLE_A group relative to the SLE_S group. Technical issues and the use of an independent cohort may have resulted in poor correlation between the 4D-LFQ and PRM data obtained for these specific phosphosites.

**Fig. 7:**
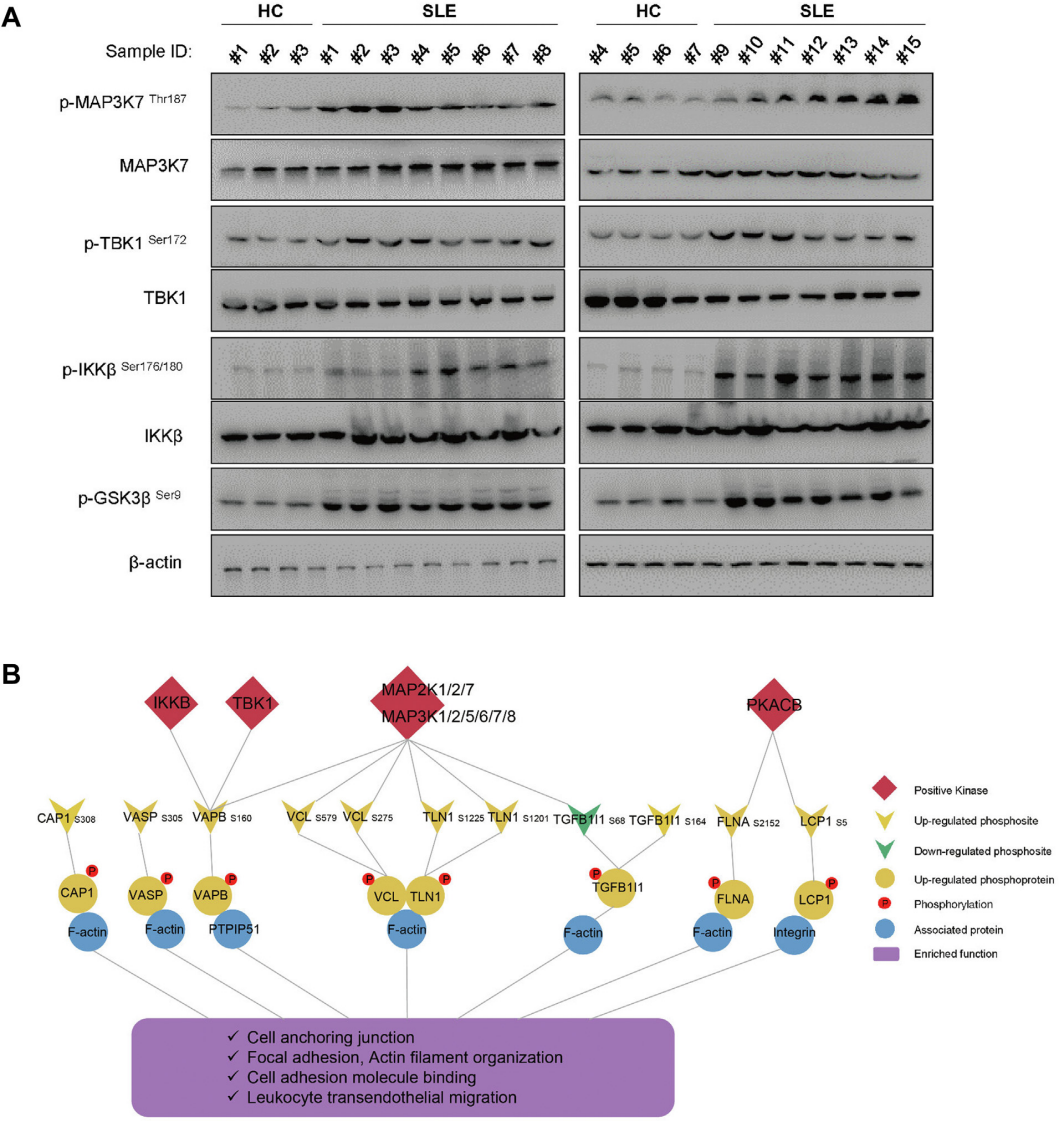
**Kinase-phosphosite pathway involved in SLE**. *A*, predicted kinases were validated by Western blot approach in SLE and HC PBMC groups. *B*, kinase-phosphosite pathway network analysis of SLE. HC, healthy control; SLE, systemic lupus erythematosus; PBMC, peripheral blood mononuclear cell; MAP3K, mitogen-activated protein kinase kinase kinase; MAP2K, mitogen-activated protein kinase kinase; TLN1, talin-1; VCL, vinculin.

We further validated some of the core predicted kinases by comparing the modification levels to the total protein levels at HC and SLE PBMC groups by Western blot approach. As shown in Fig. 7*A* in our revised manuscript, there was a marked increase in phosphorylation of MAP3K7 T187, TBK1 S172, IKKβ S176/180, and GSK3β S9 in SLE compared with those in HC groups, suggesting an increased activity of these kinases in SLE. Finally, we summarized a prominent kinase-phosphosite pathway profile in SLE according to the PRM and 4D-LFQ data. The phosphoproteomic data demonstrated the regulation of several kinases and phosphoprotein-related pathways in SLE. The network showed that the increased phosphorylation of VCL and TLN1 and decreased phosphorylation of TGFB1I1 were regulated by MAP3Ks and MAP2Ks, phosphorylation of VAPB was regulated by IKKB, TBK1, MAP3Ks, and MAP2Ks, while phosphorylation of FLNA and LCP1 was regulated by PKACB. All of these phosphorylated proteins are involved in several processes related to cell adhesion and migration, such as cell adherens junctions, focal adhesion and actin filament organization. These processes contribute to signal transduction and reorganization of the actin cytoskeleton and ultimately promote leukocyte transendothelial migration (Fig. 7*C*).

## Discussion

SLE is a complex disease featuring diverse clinical features and laboratory abnormalities, the disease progression of which is influenced by both genetic and environmental factors. Over the past decades, more than 100 genetic loci involved in SLE pathogenesis have been detected (19), but the effect of each locus on risk is relatively small. Besides the heterogeneity of genetic background, the disease activity can vary with the exposure of environmental risk factors (20). Thus, instead of exploring the potential targets at the static genetic level, our study aims to verify the aberrant dynamic cellular signal pathways in patients with SLE by performing high-throughput phosphoproteomic analyses with PBMCs obtained from HC and patients with SLE_S, SLE_A, and RA. In this study, we identified significantly regulated phosphosites and pathways associated with cell adhesion and migration in the SLE patients compared with HC and those with RA. Expression pattern clustering analysis revealed distinct phosphosites and functions between the SLE_A and SLE_S groups. In addition, key kinases were predicted, and kinase-regulated networks were built in SLE. Finally, using PRM and Western blot, we validated several phosphosites and kinases related to cell adhesion and migration in SLE. For the migration and infiltration of immune cells, they are important drivers initiating and amplifying uncontrolled tissue damage, and these phosphosites and kinases can provide potential target for the control of inflammation and ameliorating organ damage.

Previous studies have indicated that transendothelial migration is a critical step that regulates the movement of lymphocytes from blood into inflamed tissues (21), which involves a complex signaling cascade mediated by selectins, integrins, chemokines, and adhesion molecules, etc. (22). By comparing the distribution of phosphosites in SLE_S and SLE_A patients with RA patients or with HC, a series of dysregulated phosphorylation events involved in cell migration, such as actin filament organization, cell−substrate junctions, and chemotaxis regulation, were found in the SLE_S and SLE_A groups, and phosphoproteins involved in focal adhesion and leukocyte transendothelial migration were more enriched in the SLE_A group than SLE_S group. All these functions and components can promote the recruitment of leukocytes to the site of inflammation (23, 24). Using PRM, we further validated several phosphosites associated with these pathways, such as CAP1 S308, LCP1 S5, TLN-1 S1201, VCL S579, VCL S275, TGFB1I1 S68 and 164, and CD226 S290. Focal adhesions are large protein complexes that can regulate signaling between the extracellular matrix and interacting cells, affecting cell migration and cell differentiation, where TLN1 and VCL are core components because they connect integrin at the plasma membrane with the actomyosin cytoskeleton. TLN1 and VCL interbanding with F-actin is the basis for the formation of focal adhesions (25, 26). CAP1 is an actin-binding protein that can form complexes with focal adhesion kinase and talin of which the function in regulating the actin cytoskeleton and cell adhesion has been identified (27, 28). TGFB1I1, a focal adhesion scaffold protein (also known as Hic-5), has been demonstrated that tyrosine phosphorylation of TGFB1I1 can regulate lamellipodia formation to affect cell motility (29). L-plastin (LCP1) is an actin-bundling protein in the hematopoietic-specific α-actin family participating the formation of integrin-associated adhesion structures (30). It has been shown that LCP1 is crucial for regulating T-cell motility and T-cell activation (31). Since the function of these actin-bundling proteins in regulating cell adhesion and migration has been shown by a large number of studies, the phosphosite analyses in our results will help to further unravel the global regulatory network in leukocytes migration. In addition, the phosphosites of corresponding actin-bundling proteins can also provide potential therapeutic target for developing drugs to inhibit immune cell migration.

In addition, we found several upregulated phosphosites in the SLE_A group relative to the SLE_S group, such as VASP S305, ASAP1 S1041, H1-4 T18, and H1-5 S18, among which VASP can link actin filaments to invadopodia and focal adhesions, which is also crucial for promoting cell migration (32). It has been shown that VASP phosphorylation can increase podocyte motility and act as a biomarker for disease activity measurement in focal segmental glomerulosclerosis (33). By modulating signaling at the actin cytoskeleton, VASP is involved in the stabilization of endothelial barrier. Since most SLE patients also have kidney damage at different severities, exploring the function of VASP S305 in SLE activity may also be worthwhile. ASAP1 is a multidomain GTPase-activating protein for ADP-ribosylation factor-type GTPases. Several studies have demonstrated that ASAP1 can affect integrin adhesion, actin cytoskeleton, and invasion and metastasis of cancer cells (34). In addition, anti-histone antibodies were frequently detected in SLE patients, which reported to be correlated with the disease activity (35). The modification of histone is also reported to facilitate the production of antihistone antibodies (36). Taken together, by comparing SLE_A with SLE_S, our phosphoproteomic analyses can provide information for potential targets for reducing the SLE disease activity.

Interesting, our results revealed the key upstream kinase for phosphosites. MAP3Ks and MAP2Ks were found to be more activated in patients with SLE (SLE_S and SLE_A) compared with HC and those with RA. Mitogen-activated protein kinase cascades include at least three kinase families of MAP3Ks, MAP2Ks, and MAPKs. Members of these three kinase families are highly conserved across eukaryotic species and have important physiological/pathological functions, such as cell growth, differentiation, adhesion, stress, and inflammatory response (37). MAPKs can positively regulate the production of inflammatory mediators such as tumor necrosis factor, interleukin-1β, and IL-6, and their impact in T cell development and activation have also been well reported, implicating MAPKs can play a role in the pathogenesis of autoimmune disease (38, 39). A recent study revealed increased phosphorylation of several MAPK kinases and associated markers in multiple sclerosis (40). And according to the prominent kinase-phosphosite pathway profile, we summarized the data from the PRM and 4D-LFQ, MAP3Ks, and MAP2Ks are associated with increased phosphorylation of VCL, LCP1, TGFB1I1, and VAPB, indicating the MAPK pathway can affect cell adhesion and cell migration, which also provides a potential new regulatory mechanism for SLE pathogenesis study. In addition, our analysis also found IKKB, TBK1, and GSK3B were strongly activated in patients with SLE but not in HC. IKKB and TBK1 both involved in the transcription activation induced by NF-κB pathway activation, which was related with the production of inflammatory cytokines (41, 42). And GSK3B was a key regulator in control of glucose homeostasis, by which it can also participate in the development and function of immune cells (43). Based on our result, we found IKKB and TBK1 kinase activity are linked to VAPB S160 phosphorylation, which also provide a new possible mechanism for the leukocyte mobility.

Above all, by performing global phosphoproteomic analysis in large number of clinical samples, our study provides rich information to understand aberrant activation of dynamic pathway in SLE patients. Furthermore, we also explored the differential phosphorylation events correlated with disease activity. By analyzing the kinase-phosphosite correlation, we summarized several potential kinase-phosphosite pathways in SLE, by which raveling of new mechanism of SLE pathogenesis might be facilitated and new clinical strategies could be developed.

## Data Availability

The phosphoproteomics and PRM data generated in this study have been uploaded to ProteomeXchange under accession no. PXD025559 and pXD025399, respectively. Visualization of MS/MS spectra can be accessed using the following URL: https://msviewer.ucsf.edu/prospector/cgi-bin/mssearch.cgi?report_title=MS-Viewer&search_key=wvw7pyeqyq&search_name=msviewer. All the information about this manuscript has been summarized in Supplementary data, and all data are available for the corresponding author upon reasonable request. For the code of analyzing and generating the data, we have deposited it in Zenodo (https://zenodo.org/record/6881019#.Ytpka8iVVMg).

## Supplemental data

This article contains supplemental data.

## Conflict of interest

All authors declare no competing interests.

## Figures and Tables

**Supplementary Figure 1:**
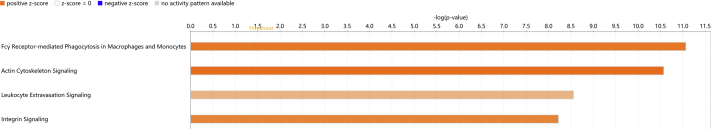
*A*, top4 enriched canonical pathway of differentially expressed phosphoproteins was shown *via* ingenuity pathway analysis (IPA) in SLE. *B*, all enriched canonical pathways of differentially expressed phosphoproteins were shown *via* IPA in SLE. *C*, activated actin signaling pathway was shown based on z-score and IPA ingenuity knowledge database, positive z-score, orange (z-score>2); the fuchsia outer ring represented the enriched proteins in this pathway. *Red* represented upregulated phosphoproteins. SLE, systemic lupus erythematosus.

**Supplementary Figure 1B:**
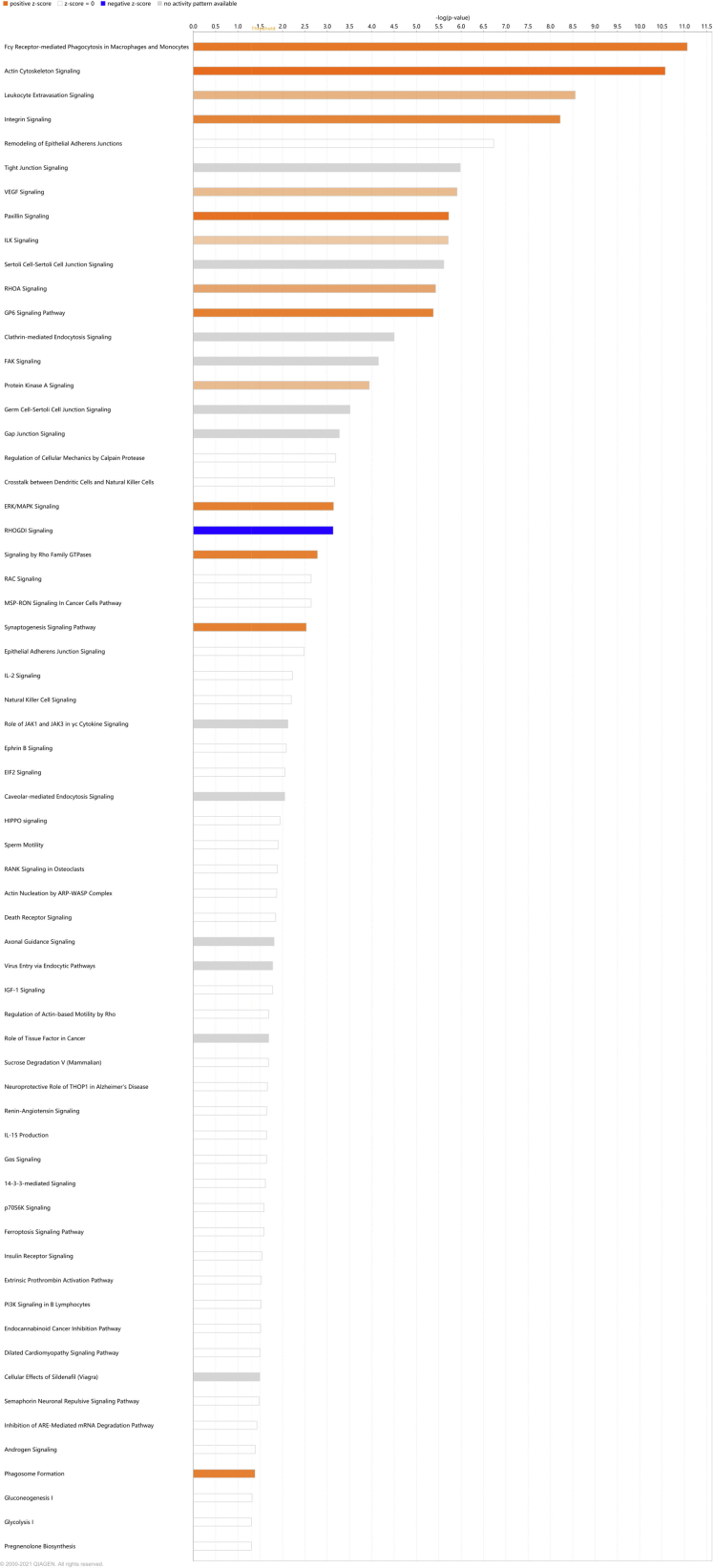


**Supplementary Figure 1C:**