# Supplementary material for: Global Phosphoproteomics Unveils Kinase-Regulated Networks in Systemic Lupus Erythematosus
Source: Mol Cell Proteomics. 2022 Oct 27;21(12):100434. doi: 10.1016/j.mcpro.2022.100434 (PMC9712766; doi:10.1016/j.mcpro.2022.100434)
Supplement: Supplementary table X [file mmc11.docx]

**Global Phosphoproteomics** **Unveils Kinase-regulated Networks in** **Systemic Lupus Erythematosus**

Shuhui Meng^1,2#^, Teng Li^1#^, Tingting Wang^2^, Dandan Li^1^, Jieping Chen^1^, Heng Li^2^, Wanxia Cai^1^, Zhipeng Zeng^1^, Donge Tang^1^*, Xiaoping Hong^2^*, Yong Dai^1*^

**Supplementary Table 1A**

|  | SLE_S | SLE_A |
| --- | --- | --- |
| Number of Subjects | 82 | 48 |
| Age (years, mean (SD)) | 40.4±12.3 | 37.2±13.4 |
| Sex, Female | 70 (85.4) | 40 (83.3) |
| **Clinical Criteria:** |  |  |
| Rash | 18 (22.0) | 14 (29.2) |
| Oral ulcers | 0 (0) | 3 (6.3) |
| Nonscarring alopecia | 2 (2.4) | 3 (6.3) |
| Synovitis involving two or more joints | 8 (9.8) | 19 (39.6) |
| Serositis | 3 (3.7) | 2 (4.2) |
| Renal disorder | 14 (17.1) | 32 (66.7) |
| Neurologic disorder | 0 (0) | 0 (0) |
| Leukopenia (< 4000/mm3 at least once) | 7 (8.5) | 4 (8.3) |
| Thrombocytopenia (<100,000/mm3) at least once | 2 (2.4) | 6 (12.5) |
| **Immunological Criteria:** |  |  |
| Anti-dsDNA | 26 (31.7) | 31 (64.6) |
| Anti-Sm | 22 (26.8) | 15 (31.3) |
| Anti-β2GPI | 5 (6.1) | 3 (6.3) |
| ACL-IgG | 8 (9.8) | 5 (10.4) |
| ACL-IgM | 1(1.2) | 2 (4.2) |
| Low complement (low C3) | 22 (26.8) | 29 (60.4) |
| Low complement (low C4) | 25 (30.5) | 22 (45.8) |
| Direct Coombs test in the absence of hemolytic anemia | 9 (11.0) | 10 (20.8) |

Data are positive rate shown as number (%) excluding some patients with missing data. NA: not applicable.

**Supplementary Table 1B**

|  | RA | HC |
| --- | --- | --- |
| Number of Subjects | 96 | 90 |
| Age (years, mean (SD)) | 50.6±15.0 | 39±10.5 |
| Sex, Female (% of samples) | 82 (85.4) | 76 (84.4) |
| Disease duration years, (mean (SD)) | 10.0±8.7 | NA |
| SJC(mean (range)) | 4 (0-28) | NA |
| TJC(mean (range)) | 6 (0-28) | NA |
| ESR (mm/hour, mean (SD)) | 35.6±27.2 | NA |
| CRP (mg/l, mean (SD)) | 21.2±31.9 | NA |
| RF positive (%) | 47 (49.0) | NA |
| CCP positive (%) | 48 (50.0) | NA |
| DAS28 score (mean (SD)) | 4.2±1.6 | NA |

Data are shown excluding some patients with missing data. SJC, swollen joint count; TJC, tender joint count; ESR, erythrocyte sedimentation rate; CRP, C-reactive protein; CCP, anti-cyclic citrullinated peptide antibody; DAS28, Disease Activity Score (28-joint count); RF, rheumatoid factor. NA: not applicable.

**Clinicopathologic characteristics of the patients for Parallel Reaction Monitoring (PRM).**

**Supplementary Table 2A**

|  | SLE_S | SLE_A |
| --- | --- | --- |
| Number of Subjects | 12 | 7 |
| Age (years, mean (SD)) | 41.6±8.4 | 40.5±9.3 |
| Sex, Female | 11(91.7) | 6 (85.7) |
| **Clinical Criteria:** |  |  |
| Rash | 4 (33.3) | 4 (57.1) |
| Oral ulcers | 0 (0) | 1 (14.3) |
| Nonscarring alopecia | 0 (0) | 1 (14.3) |
| Synovitis involving two or more joints | 2 (16.7) | 4 (57.1) |
| Serositis | 1 (8.3) | 1 (14.3) |
| Renal disorder | 2 (16.7) | 5 (71.4) |
| Neurologic disorder | 0 (0) | 0 (0) |
| Leukopenia (< 4000/mm3 at least once) | 2 (16.7) | 3 (42.9) |
| Thrombocytopenia (<100,000/mm3) at least once | 0 (0) | 0 (0) |
| **Immunological Criteria:** |  |  |
| Anti-dsDNA | 6 (50.0) | 4 (57.1) |
| Anti-Sm | 3 (25.0) | 1 (14.3) |
| Anti-β2GPI | 1 (8.3) | 0 (0) |
| ACL-IgG | 2 (16.7) | 2 (28.6) |
| ACL-IgM | 0 (0) | 1 (14.3) |
| Low complement (low C3) | 4 (33.3) | 4 (57.1) |
| Low complement (low C4) | 4 (33.3) | 4 (57.1) |
| Direct Coombs test in the absence of hemolytic anemia | 1(8.3) | 2 (28.6) |

Data are positive rate shown as number (%) excluding some patients with missing data. NA: not applicable.

**Supplementary Table 2B**

|  | RA | HC |
| --- | --- | --- |
| Number of Subjects | 10 | 8 |
| Age (years, mean (SD)) | 46.6±12.0 | 44.0±10.5 |
| Sex, Female (% of samples) | 8 (80.0) | 7 (87.5) |
| Disease duration years, mean (SD)) | 12.1±6.6 | NA |
| SJC(mean (range)) | 4 (0-28) | NA |
| TJC(mean (range)) | 4 (0-28) | NA |
| ESR (mm/hour,mean (SD)) | 30.6±26.9 | NA |
| CRP (mg/l,mean (SD)) | 25.5±36.1 | NA |
| RF positive (%) | 5 (50.0) | NA |
| CCP positive (%) | 4 (40.0) | NA |
| DAS28 score (mean (SD)) | 4.2±1.2 | NA |

Data are shown excluding some patients with missing data. SJC, swollen joint count; TJC, tender joint count; ESR, erythrocyte sedimentation rate; CRP, C-reactive protein; CCP, anti-cyclic citrullinated peptide antibody; DAS28, Disease Activity Score (28-joint count); RF, rheumatoid factor. NA: not applicable.

**Clinicopathologic characteristics of the patients for Western blot**

**Supplementary Table 3**

|  | SLE_S | SLE_A | HC |
| --- | --- | --- | --- |
| Number of Subjects | 6 | 9 | 7 |
| Age (years, mean (SD)) | 36.7±11.2 | 42.8±13.5 | 39.7±12.5 |
| Sex, Female | 6 (100.0) | 9 (100.0) | 7 (100.0) |
| **Clinical Criteria:** |  |  |  |
| Rash | 1 (16.7) | 3 (33.3) | NA |
| Oral ulcers | 0 (0) | 0 (0) | NA |
| Nonscarring alopecia | 2 (33.3) | 2 (22.2) | NA |
| Synovitis involving two or more joints | 1 (16.7) | 3 (33.3) | NA |
| Serositis | 1 (16.7) | 0 (0) | NA |
| Renal disorder | 0 (0) | 4 (44.4) | NA |
| Neurologic disorder | 0 (0) | 0 (0) | NA |
| Leukopenia (< 4000/mm3 at least once) | 1 (16.7) | 0 (0) | NA |
| Thrombocytopenia (<100,000/mm3) at least once | 1 (16.7) | 1 (11.1) | NA |
| **Immunological Criteria:** |  |  |  |
| Anti-dsDNA | 3 (50.0) | 8 (88.9) | NA |
| Anti-Sm | 3 (50.0) | 3 (33.3) | NA |
| Anti-β2GPI | 0 (0) | 2 (22.2) | NA |
| ACL-IgG | 0 (0) | 2 (22.2) | NA |
| ACL-IgM | 0 (0) | 1 (11.1) | NA |
| Low complement (low C3) | 5 (83.3) | 7 (77.8) | NA |
| Low complement (low C4) | 3 (50.0) | 7 (77.8) | NA |
| Direct Coombs test in the absence of hemolytic anemia | 0 (0) | 1 (11.1) | NA |

Data are positive rate shown as number (%) excluding some patients with missing data. NA: not applicable.
