# Supplementary material for: Global Phosphoproteomics Unveils Kinase-Regulated Networks in Systemic Lupus Erythematosus
Source: Mol Cell Proteomics. 2022 Oct 27;21(12):100434. doi: 10.1016/j.mcpro.2022.100434 (PMC9712766; doi:10.1016/j.mcpro.2022.100434)
Supplement: Supplementary Tables [file mmc12.docx]

**Supplementary Tables：**

Supplementary table 1: Differentially expressed phosphosites and phosphoproteins for comparable groups

Supplementary table 2: Venn table of differential and overlapping phosphosites for comparable groups

Supplementary table 3: Enrichment of Gene Ontology (GO) analysis for comparable groups

Supplementary table 4: Enrichment of KEGG pathway analysis for comparable groups

Supplementary table 5: Phosphosites of 5 clusters

Supplementary table 6: Normalized enrichment scores (NESs) of kinase activity for HC, SLE_S, SLE_A and RA

Supplementary table 7: Parallel Reaction Monitoring (PRM) result of validated phosphosites for HC, SLE_S, SLE_A and RA

Supplementary table 8: Phosphoproteins that were used to perform IPA

Supplementary table A: All phosphopeptide sequences for phosphoproteomics

Supplementary table B: Validated phosphopeptide sequences for PRM

Supplementary table X: Clinical information for HC, SLE_S, SLE_A and RA
